# Automatic Quantitative Assessment for Diagnostic and Therapeutic Response in Rodent Myocardial Infarct Model

**DOI:** 10.3390/biomedicines12010219

**Published:** 2024-01-18

**Authors:** Kangsan Kim, Yong Jin Lee, Min Hwan Kim, Byung Hyun Byun, Sang-Keun Woo

**Affiliations:** 1 Division of Applied RI, Korea Institute of Radiological and Medical Sciences, Seoul 01812, Republic of Korea; 2 Research Institute of Radiopharmaceuticals, FutureChem Co., Ltd., Seoul 04794, Republic of Korea; 3 Department of Nuclear Medicine, Korea Institute of Radiological and Medical Sciences, Seoul 01812, Republic of Korea

**Keywords:** myocardial infarction, deep learning, Otsu’s algorithm, segmentation, positron emission tomography, single photon emission computed tomography, magnetic resonance image

## Abstract

The purpose of this study was to investigate the most appropriate methodological approach for the automatic measurement of rodent myocardial infarct polar map using histogram-based thresholding and unsupervised deep learning (DL)-based segmentation. A rat myocardial infarction model was induced by ligation of the left coronary artery. Positron emission tomography (PET) was performed 60 min after the administration of ^18^F-fluoro-deoxy-glucose (^18^F-FDG), and PET was performed after injecting ^64^Cu-pyruvaldehyde-bis(N4-methylthiosemicarbazone). Single photon emission computed tomography was performed 60 min after injection of ^99m^Tc-hexakis-2-methoxyisobutylisonitrile and ^201^Tl. Delayed contrast-enhanced magnetic resonance imaging was performed after injecting Gd-DTPA-BMA. Three types of thresholding methods (naive thresholding, Otsu’s algorithm, and multi-Gaussian mixture model (MGMM)) were used. DL segmentation methods were based on a convolution neural network and trained with constraints on feature similarity and spatial continuity of the response map extracted from images by the network. The relative infarct sizes measured by histology and estimated R^2^ for ^18^F-FDG were 0.8477, 0.7084, 0.8353, and 0.9024 for naïve thresholding, Otsu’s algorithm, MGMM, and DL segmentation, respectively. DL-based method improved the accuracy of MI size assessment.

## 1. Introduction

Quantitative analysis of myocardial infarction (MI) size is necessary to reduce patient mortality and assess therapeutic effects in cardiac disease research [1,2,3,4]. Cardiac disease research for improving diagnosis and therapy is validated extensively using nuclear medicine imaging. Anatomical and functional images in cardiac disease are non-invasive evaluation modalities [5,6]. Single photon emission computed tomography (SPECT) is used to assess myocardial viability and perfusion using ^99m^Tc-hexakis-2-methoxyisobutylisonitrile (^99m^Tc-MIBI) and carrier-free ^201^Tl [7,8,9]. A positron emission tomography (PET) study is used to evaluate myocardial viability and perfusion accurately using ^18^F-fluoro-deoxy-glucose (^18^F-FDG), which evaluates the rate of glucose uptake in the myocardium, and ^64^Cu-pyruvaldehyde-bis(N4-methylthiosemicarbazone) (^64^Cu-PTSM), which is used as a blood flow tracer [10,11]. Advancements in medical technology have enabled more accurate evaluations of myocardial perfusion and MI size using delayed contrast-enhanced magnetic resonance imaging (DCE-MRI) and computed tomography (CT) [12,13]. MI size is evaluated using MRI using contrast agents containing gadolinium to acquire the high-resolution DCE-MR images, and CT is used with iodixanol to acquire high-resolution DCI CT images.

Multimodal imaging methods are used to analyze myocardial characteristics of small animals such as rats with and without MI using PET, SPECT, and MRI; however, evaluation of cardiac function using these methods is difficult because of acquisition by different modalities. The quantitative assessment of the MI area is also compromised because of low image quality and distortion of the location information due to heart movements. In addition, the degree of ^18^F-FDG uptake in the apex of the heart is lower in the polar map obtained through small animal PET [14]. For these reasons, research in cardiac disease for methods to overcome limitations in the evaluation of MI size is required.

The traditional assessment methods for the MI area used various predefined thresholds applied to polar maps [15,16,17,18,19]. Some studies used the automated feature analysis and combined threshold (FACT) as a computer algorithm with intensity histogram and threshold value [20]. Non-invasive evaluation technologies measured MI size using segment software after manual and automated delineations on histological data and ^18^F-FDG PET images [21]. However, automation of quantitative analysis of MI size is difficult despite these achievements. Various automatic algorithms for the assessment of cardiovascular MR images are available [19]. Additionally, the comparison with methods using predefined absolute values and hematoxylin and eosin (H&E) staining method has shown a difference in measured MI size between nuclear medicine and histological images.

Rather than the histogram-based pixel classification introduced above, most recent studies on image segmentation have adopted the convolution neural network (CNN), which extracts the representation of a given image based on local texture [22,23,24]. While many deep learning (DL)-based segmentation studies in medical imaging have shown remarkable results, the application of DL in MI area estimation using polar maps is controversial. This is because polar map images lack structural information compared to anatomical images. Moreover, due to the difficulty in obtaining the ground truth segmentation map, unsupervised segmentation methods should be considered.

In this study, we evaluated the MI area in polar maps obtained using multimodal imaging for quantitative assessment of MI size using histogram-based pixel threshold methods, including multi-Gaussian mixture model (MGMM) and DL-based unsupervised segmentation methods.

## 2. Materials and Methods

### 2.1. MI Modeling

Eleven white male Sprague-Dawley (SD) rats of the age of 8 months were used for quantitative assessment of small animal MI size. All rats were anesthetized using 2% isoflurane (Forane Solution) while breathing 100% oxygen with a respirator to obtain MI disease models. The chest was opened to expose the heart, and the left coronary artery (LCA) was ligated permanently on a heating pad. PET imaging was performed on rats in the MI model group 3 days after ligation for assessment of myocardial viability and perfusion. For assessment of MI disease modeling, DCE-MRI that evaluates ventricular structure with a high resolution was performed after PET imaging. Each MI model rat was provided with adequate food and housed in a warm environment that was maintained during preprocessing [25].

### 2.2. Rat PET/SPECT/MRI Acquisition

The PET/SPECT images were obtained with a small animal PET/SPECT scanner (InveonTM, Siemens, Munich, Germany). Rat cardiac gate signals were analyzed using the external trigger BioVET (m2m Imag. Corp., Rock Island, IL, USA), and the trigger signals were recorded with the event signal. PET imaging was performed 60 min after the administration of 30 MBq of ^18^F-FDG, and a dynamic PET scan was performed after the injection of 37 MBq of ^64^Cu-PTSM via a tail vein injection. The Inveon scanner, which has Lutetium Oxyorthosilicate scintillation crystals, was used as the small animal PET scanner and acquired 3 spans and 31 ring differences through a setting in the 350–650 keV energy window. The acquired list mode data were reconstructed using the ordered subset expectation maximization 2D (OSEM 2D) algorithm with four iterations, after being converted to sinogram by the Fourier Rebinning method using a trigger signal. SPECT images were acquired 60 min after the injection of 110 MBq of ^99m^Tc-MIBI and ^201^Tl/0.1 mL via the tail vein with the following settings: 126–154 keV (Tc), 63–77 keV (Tl) energy window, 35 mm radius of rotation, 60 s/angle, and 1.0 mm pinhole collimator. SPECT images were reconstructed using the OSEM 3D algorithm with 4 subsets and 8 iterations. MR images for confirming MI modeling were obtained using a 3-T clinical MRI scanner (MAGNETOM Tim Trio, Siemens) with mobile electrocardiogram triggering at 2 days after ligation. For contrast enhancement of the MI region, we used Gd-DTPA-BMA (Omniscan) 0.5 mmol/kg injection. DCE images were obtained with a T1-weighted turbo flash PSIR 2D sequence 10 min after the injection of a contrast agent. In total, eleven ^18^F-FDG PET, three ^64^Cu-PTSM PET, five ^99m^Tc-MIBI SPECT, and four ^209^Tl SPECT images were acquired.

### 2.3. Polar Map Generation and Left Ventricle Staining

The rat PET/SPECT cardiac images were implemented to match the pixel size of the images of heart images of actual patients similarly by multiplying the scaling factor [26].

Inveon PET/SPECT images of the cardiac area were converted to Digital Imaging and Communications in Medicine format, which can be read by other image processing software following rotation and cropping. The resampling images of the cardiac area were generated in a short-axis direction, horizontal long axis, and vertical long axis and converted to polar map images using the Clinical QGS software (Cedars QGS 2008, Syngo, Siemens), which can set the MI area automatically for the evaluation of rat PET/SPECT cardiac images. The rat hearts were harvested under anesthesia with 5% isoflurane inhalation for assessment of visible myocardium and then stained [27]. Left ventricles were sectioned into 10 µm slices, and five slices (50 µm) were stained using H&E. The rat MI size was assessed as the ratio of the MI area to the entire area of the myocardium.

### 2.4. Automatic Quantitative Analysis of Polar Maps

MI size is calculated as the percentage of the ratio of the MI area that is below the threshold value to the entire area of the polar map. The threshold value was implemented using the absolute value setting method, Otsu method algorithm, and MGMMs. In the absolute value method, the nine threshold values were set to 10–90% divided by 10 percent unit. The Otsu algorithm is widely used to set the threshold value using the statistical characteristics of the histogram that appears in the distribution of pixel brightness in image binarization. It sets an arbitrary threshold value to delineate a similar distribution of pixel values between normal myocardial regions and infarct areas in the polar map. The threshold value determined using the Otsu method maximized the distribution of two areas with low within-class variance [28].

GMMs were widely employed for using statistical characteristics such as pattern recognition and image segmentation. The pixel intensity histogram of the MI polar map image is composed of lower and upper peaks, which correspond to the infarct region and normal myocardial region, respectively. The distribution of the entire polar map image value according to the probability density function, which is the statistical distribution, can be represented as the GMM in the MI areas and normal myocardial regions, as shown in (1). For the input given pixel intensity *x*, the GMM model is expressed as the linear combination of the Gaussian basis function, where *j*th basis function is denoted as $f\left(x|\psi_{j}\right)$ in (1). *π_j_* represents the weight of each Gaussian basis. Parameters of the *j*th Gaussian distribution, average and variation, are denoted as *μ_j_* and *σ_j_*^2^, respectively [29]. The parameterization of the Gaussian basis is described in (2).

(1) $$f\left(x|\psi \right)=\sum_{j=1}^{N}\pi_{j}f\left(x|\psi_{j}\right)$$

(2) $$f\left(x|\psi_{j}\right)=f\left(x_{i}|\mu_{j},\sigma_{j}^{2}\right)=\frac{1}{\sqrt[{}]{2\pi }\sigma_{j}}\exp\left\{-\frac{{\left(x_{i}-\mu_{j}\right)}^{2}}{2\sigma_{j}^{2}}\right\}$$

The MGMM method was designed for automatic quantitative polar map analysis. The aim was to generate multiple Gaussian basis functions based on the expectation–maximization (EM) algorithm. In iteration, $\omega_{j}^{i(k)}$, the probability of whether pixel k belongs to the *j*th Gaussian basis is updated to attain the maximum convergence of log likelihood. It is expressed as the fraction of the *j*th Gaussian basis, as expressed in (3). It is updated using Formulae (4) and (5) in each iteration. In the formula, *L* represents the number of data, and *k* is the number of iterations of the EM algorithm. The MGMM, which is suitable for MI polar map analysis, improves the performance of calculating the threshold value because the first average value can be calculated from the preceding Gaussian function using (3). MGMM values were obtained by estimating the weights, probability, and variance as parameter values determined by (*k* + 1)th in Formula (4) after calculating the whole probability of number *j* Gaussian functions.

(3) $$\omega_{j}^{i(k)}=\frac{\pi_{j}^{\left(k\right)}f\left(x_{i}\left|\mu_{j}^{\left(k\right)},\sigma_{j}^{\left(k\right)}\right.\right)}{\sum_{l=1}^{L}\pi_{l}^{\left(k\right)}f\left(x_{i}\left|\mu_{l}^{\left(k\right)},\sigma_{l}^{\left(k\right)}\right.\right)}\;$$

(4) $$S_{j}=\frac{1}{2}\sum_{a=1}^{j}\omega_{a}^{i(k)}$$

(5) $$\omega_{j}^{i(k+1)}=\left(1-S_{j-1}\right)\frac{\pi_{j}^{\left(k\right)}f\left(x_{i}\left|\mu_{j}^{\left(k\right)},\sigma_{j}^{\left(k\right)}\right.\right)}{\sum_{l=1}^{L}\pi_{l}^{\left(k\right)}f\left(x_{i}\left|\mu_{l}^{\left(k\right)},\sigma_{l}^{\left(k\right)}\right.\right)}$$

After determining the probability of belonging to the *j*th Gaussian basis, the weight of the basis and the parameter of each Gaussian basis are updated as (6)–(8).

(6) $$\pi_{j}^{\left(k+1\right)}=\frac{1}{N}\sum_{i=1}^{N}\omega_{j}^{i(k+1)}$$

(7) $$\mu_{j}^{\left(k+1\right)}=\frac{\sum_{i=1}^{N}\omega_{j}^{i(k+1)}x_{i}}{\sum_{i=1}^{N}\omega_{j}^{i(k+1)}}$$

(8) $${\left\{\sigma_{j}^{2}\right\}}^{\left(k+1\right)}=\frac{1}{\sum_{i=1}^{N}\omega_{j}^{i(k+1)}}\sum_{i=1}^{N}\omega_{j}^{i(k+1)}{\left\{x_{i}-\mu_{j}^{\left(k+1\right)}\right\}}^{2}$$

The MGMM method can be used to differentiate between normal and MI areas in the polar map using optimal parameters.

In this method, the number of Gaussian basis functions is the hyperparameter to be adjusted. For comparison, we denoted the MGMM model with the *j*th Gaussian basis as MGMM(*j*). The threshold value was the overlapping point of the first and second Gaussian functions using the characteristic of the infarct area with a low pixel intensity. The MI area of small animal images was determined by the evaluated threshold value using each method and compared to the H&E stained area obtained histologically as the standard size.

Moreover, we also used the CNN-based unsupervised segmentation model proposed by Kim et al. [30] and assessed the method for MI area segmentation. In the method, the number of pixel clusters and the label of pixels were adaptively determined by iteration. For each iteration, the CNN outputs the pixel-wise classification probability map, and each pixel is classified as the label with the maximum probability. From the large number of clusters set initially, pixels with similar features or adjacent pixels were integrated according to the iteration.

The neural network F consists of feature extractor *W_F_* and classifier *W_c_*, which are composed of one fully convolutional network and batch normalization layer. In the iteration process, *F* extracts the normalized response map *r* = *F*(*x*). The loss function is expressed as the weighted sum of two terms that represent the feature similarity and spatial continuity. The feature similarity loss is the cross entropy loss between the *r* and *c*, the cluster-wise argmax label for the *r*. For the *r_i_*, the response map for *i*th cluster, the feature similarity loss *L_sim_* is expressed as (9).

(9) $$L_{sim}\left(\left\{r,c\right\}\right)=\sum_{i=1}^{q}-\delta \left(i-c\right)\ln r_{i}$$

(10) $$\delta \left(t\right)=\left\{\begin{array}{cc} 1 & if\;t=0\\ 0 & otherwise \end{array}\right.$$

Moreover, Kim et al. also considered the constraint in the spatial continuity of pixels within the clusters. The spatial continuity was calculated as the *L*1 loss between the response map *r* and its parallel translation as a unit pixel horizontally or vertically. The spatial continuity loss was defined as (11).

(11) $$L_{con}\left(\left\{r\right\}\right)=\sum_{w=1}^{W-1}\sum_{h=1}^{H-1}{\left\|r_{w+1,h}-r_{w,h}\right\|}_{1}+{\left\|r_{w,h+1}-r_{w,h}\right\|}_{1}\;$$

where *W* and *H* indicate the width and height of the given image *x* and *r_w_*_,*h*_ represents the pixel value of the response map at (*w*, *h*).

Finally, the total loss is expressed as the sum of the two loss functions introduced above.

(12) $$L=L_{sim}\left(\left\{r,c\right\}\right)+L_{con}\left(\left\{r\right\}\right)$$

After the training was completed, at least three pixel clusters were obtained for each image. To determine the MI area from the segmentation map, we calculated the cluster-wise average of pixel intensities. Since the background of the polar map was black, we selected the second lowest cluster as the MI area.

### 2.5. Evaluation

To compare the imaging agents for estimating the MI area, the histological staining results using H&E were used as the ground truth. For each radiopharmaceutical predefined threshold, the Otsu algorithm, MGMM, and CNN-based unsupervised segmentation were applied. The results were compared with the ground truth by calculating their relative size difference.

We conducted linear fitting between the ground truth and the predicted values for ^18^F-FDG for the eleven images. The coefficient of determination (R^2^) was estimated for quantitative evaluation. Moreover, a paired *t*-test was conducted between the automatic assessment methods by comparing the prediction results for ^18^F-FDG PET. The statistical test was conducted using GraphPad Prism v10.1.0 software.

## 3. Results

### 3.1. Rodent Myocardial PET/SPECT Imaging

**Figure 1:**
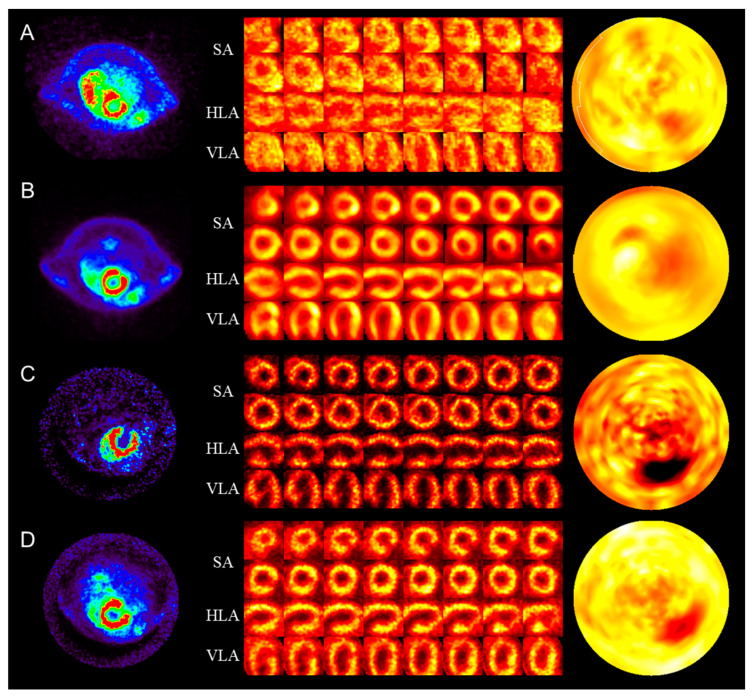
Myocardial infarction model images of various radiopharmaceuticals: (**A**) ^64^Cu-pyruvaldehyde-bis(N4-methylthiosemicarbazone); (**B**) ^18^F-fluoro-deoxy-glucose; (**C**) ^99m^Tc-hexakis-2-methoxyisobutylisonitrile; (**D**) ^201^Tl. The first column shows positron emission tomography/single photon emission computed tomography myocardial image. The second column shows the splash images SA, HLA, and VLA. The third column shows the polar map image. SA, short axis view; HLA, horizontal long axis; VLA, vertical long axis.

The right column in Figure 1 shows the rat polar map images generated from PET and SPECT images using the QGS software. We observed good sensitivity of the polar map because PET and SPECT studies were performed with proper acquisition time and activity. Small animal PET/SPECT images were acquired to evaluate viability and perfusion in MI rat models with ligated LCA. PET studies were performed by injecting ^64^Cu-PTSM and ^18^F-FDG. SPECT studies were performed by injecting ^99m^Tc-MIBI and ^201^Tl, as shown in the left column in Figure 1. MI polar maps generated for each radiopharmaceutical visualized the MI area well. The uptake of radiopharmaceuticals in the myocardium was higher than in other areas [31]; on the contrary, it was low in the left ventricle attachment area, which was suspected to be MI in each cross-sectional image. The polar map of the normal myocardium shows a relatively uniform distribution, while the polar map of the MI area in the rat model showed a low uptake at the apex. This could be because of the decreased activity in the apex by physiological apical thinning [32].

### 3.2. Evaluation of MI Area in the Polar Map

**Figure 2:**
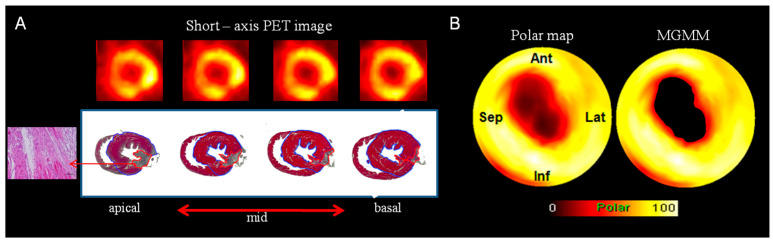
Measurement of myocardial infarct size based on polar maps: (**A**) Comparison of short axis PET image and histological section stained using hematoxylin and eosin. The histological section is shown from base to apex. The total left myocardium and infarct areas are represented by the blue and red lines, respectively. (**B**) The myocardial infarct model polar map image (**left**) and polar map image, which applied the adaptive threshold value generated using the MGMM method (**right**). PET, positron emission tomography; MGMM, multi-Gaussian mixture model.

The MI area was evaluated using the polar map and histological sections (Figure 2A).

**Figure 3:**
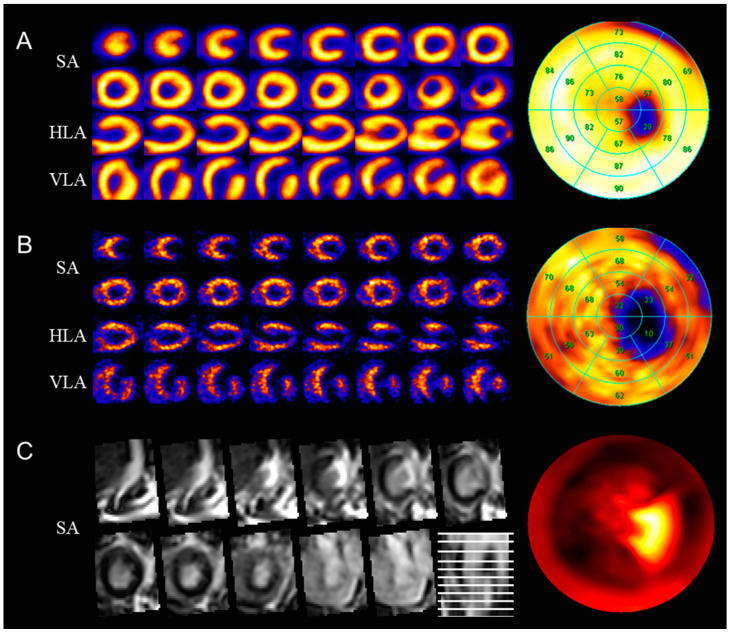
Myocardial infarction rat model PET/SPECT image compared to MR image: (**A**) ^18^F-fluoro-deoxy-glucose PET image; (**B**) ^99m^Tc-hexakis-2-methoxyisobutylisonitrile SPECT image; (**C**) delayed contrast-enhanced magnetic resonance image. Splash image SA, HLA, and VLA views (**left**) and polar map image segmented into 20 regions using the Cedars-Sinai method (**right**). PET, positron emission tomography; SPECT, single photon emission computed tomography; SA, short axis view; HLA, horizontal long axis; VLA, vertical long axis.

The total myocardium size was calculated by estimating the entire left myocardial area bordered by the blue line. Similarly, MI size was calculated by estimating the inner area of the red contour in the left myocardium dyed by H&E staining. The MI size of small animals was determined by calculating the difference between the size of the polar map and the histological sections (standard size). Three-dimensional myocardium images were converted as the polar map consisting of 20 blocks in two dimensions for easy evaluation using the Cedars-Sinai method (Figure 3) [33]. Figure 3 shows the reoriented image and segmented polar map with ^18^F-FDG PET, ^99m^Tc-MIBI SPECT, and DCE MR images. A comparison of the visualized polar map of DCE MR and function images confirmed that both can be used for evaluating the MI area. To evaluate the specific characteristics in radiopharmaceutical and contrast agents, the MI area percentages of staining for ^18^F-FDG PET, ^99m^Tc-MIBI, ^201^Tl SPECT, and DCE MRI polar maps were determined as 3.54%, 6.83%, 10.82%, 11.79%, 3.91%, respectively.

### 3.3. Comparison of Various MI Size Evaluation Methods

**Figure 4:**
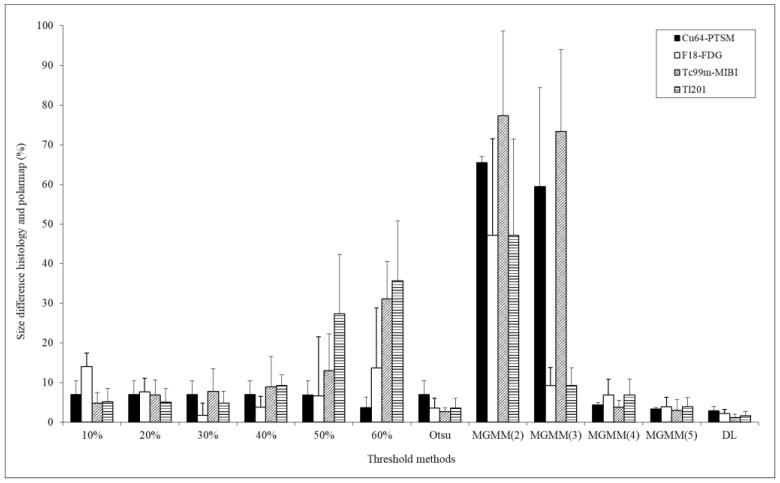
Differences in evaluated infarct size between histology and polar map in accordance with various threshold value methods. Each radiopharmaceutical image was quantitatively compared by the predefined absolute threshold value (10~60%) and the Otsu and MGMM (2, 3, 4, 5) methods, respectively. DL indicates deep learning CNN-based unsupervised segmentation method.

The differences between the obtained object models and the differences in MI size are shown in Figure 4. The comparison showed that the optimal number of the Gaussian basis in MGMM was four. Therefore, we denoted the optimal MGMM as MGMM. The difference between the infarct size measured using H&E staining and ^64^Cu-PTSM polar map was 6.97 ± 3.10% (mean ± standard deviation) in the absolute value 30%, 6.96 ± 3.53% in the absolute value 40%, and 6.93 ± 3.54% in the absolute value 50%. Otsu algorithm was 6.97 ± 3.53%, MGMM was 3.33 ± 0.41%, and DL segmentation was 2.94 ± 0.99%. The difference between the infarct size measured by H&E staining and ^201^Tl polar map was 4.79 ± 3.10% in the absolute value 30%, 9.24 ± 2.75% in the absolute value 40%, and 27.38 ± 14.91% in the absolute value 50%. Otsu algorithm was 3.59 ± 2.45%, MGMM was 3.87 ± 2.40%, and DL-segmentation was 1.60 ± 1.11%. According to the results, the DL-based segmentation method showed the least difference with the standard size stained by H&E.

**Figure 5:**
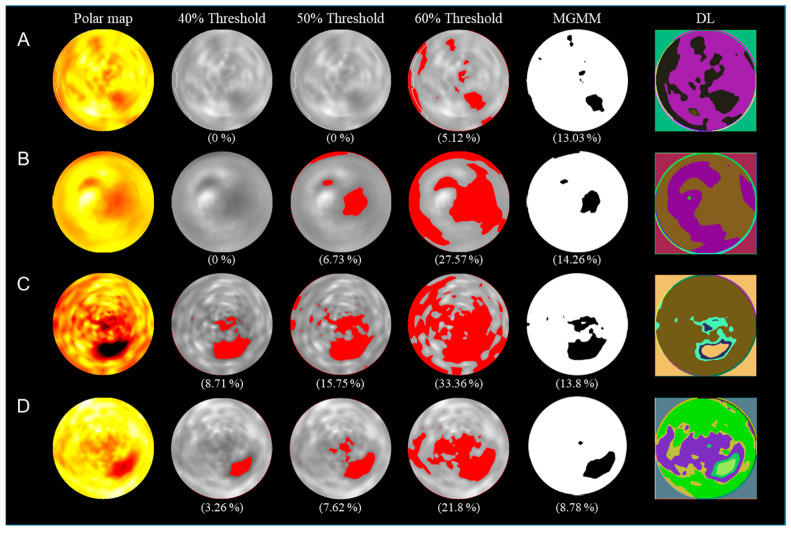
Comparison of the evaluated infarct size using various threshold value methods for each radiopharmaceutical: (**A**) ^64^Cu-PTSM; (**B**) ^18^F-FDG; (**C**) ^99m^Tc-MIBI; (**D**) ^201^Tl. DL indicates deep learning CNN-based unsupervised segmentation method.

Figure 5 shows the comparison of the evaluated infarct size using various methods. The threshold value is set using the predefined absolute threshold value method and the MGMM method on the generated polar map for the evaluation of MI size in PET/SPECT images [34]. The histologically stained H&E area used as the standard size of the MI area was 15%. The infarct sizes using the predefined 40% threshold value of ^64^Cu-PTSM, ^18^F-FDG, ^99m^Tc-MIBI, and Tl polar map were 0%, 0%, 8.71%, and 3.26%, respectively. The infarct size using the adaptive threshold values with the MGMM algorithm of ^64^Cu-PTSM, ^18^F-FDG, ^99m^Tc-MIBI, and ^201^Tl polar map were 13.03%, 14.26%, 13.8%, and 8.78%, respectively.

**Figure 6:**
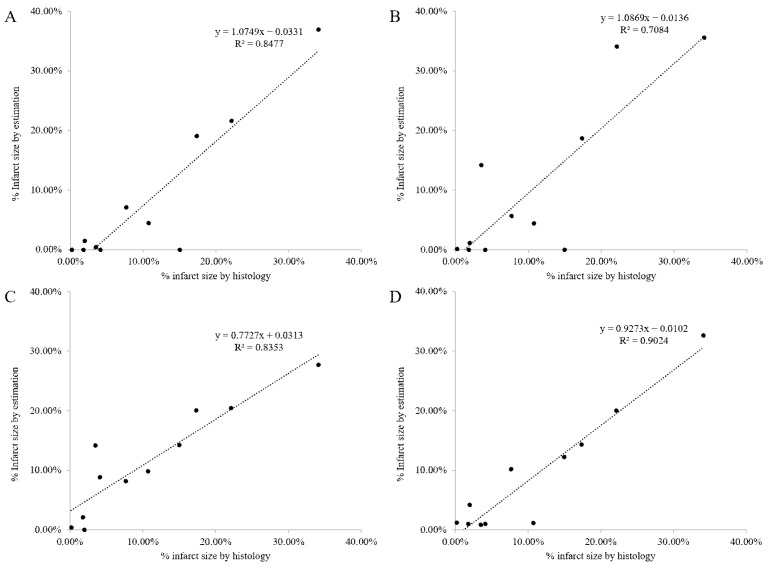
The correlation analysis of infarct size determined by histology and various threshold value methods: (**A**) 40% absolute threshold value; (**B**) Otsu algorithm; (**C**) MGMM; (**D**) DL segmentation method.

Figure 6 shows the result of the assessment of the correlation between histological images and PET polar maps in the measurement of the MI area. The predefined threshold value method and the standard size had a high correlation with an index of 0.8477 when measuring the infarct size using 40% of the absolute threshold value in the obtained polar map image from small animal PET. The correlation index of the Otsu algorithm was 0.7084, MGMM was 0.8353, and DL-based segmentation was 0.9024, which scored the highest correlation. There was a difference depending on the size of the infarct area. Paired *t*-test was conducted by comparing the DL-based segmentation and the others. According to the test, the *p* value of the 40% thresholding, Otsu algorithm, and MGMM were 0.6302, 0.5586, and 0.1827, respectively. It was found that the prediction methods were not significantly different.

## 4. Discussion

We presented the quantitative assessment of myocardial perfusion and viability in MI and the stem cell treatment effects using automatic adaptive threshold values. The PET/SPECT images of the normal rat myocardium were acquired using various radiopharmaceuticals: ^64^Cu-PTSM, ^18^F-FDG, ^99m^Tc-MIBI, and ^201^Tl. The obtained normal rat images showed sufficient sensitivity and contrast in the myocardium area; therefore, these images could be accurately converted to polar maps for quantitative evaluation. In these polar maps, the MI area was automatically segmented and contoured by the Siemens QGS software. This enabled the objective and reproducible assessment of MI size. DCE-MRI images clearly showed the myocardium area of rats due to proper contrast enhancement by Omniscan. The MRI polar maps were obtained accurately, as shown in Figure 3C, in which the MI area was defined from the segmented myocardium area. The results of the assessment of normal myocardium were sufficient to demonstrate the sensitivity, accuracy, and worth as the control group for the evaluation of MI size in each of the radiopharmaceutical modality groups.

The MI models adequately simulated the infarct area due to permanent ligation of the LCA of SD rats under anesthesia after the provision of adequate rest and food. The polar map possesses the anatomical and functional information of the MI rat; however, it was difficult to assess MI size quantitatively with the unaided eye. Therefore, a quantitative and reproducible assessment of the MI area is necessary for accurate diagnosis and treatment of cardiac disease. MI size could be accurately evaluated in polar maps of MI models using our MGMM method. The hearts of MI models were harvested from the body and then sectioned and stained using H&E after image acquisition. The assessment of MI size by histological staining determined the percentage of the infarct area in the entire left ventricle. This histological section-defined MI area is shown in Figure 2A; therefore, we had the reference value for MI assessment. The MI area indicated by H&E staining and the polar maps showed a high correlation.

Since the substructure within the polar map image is not vivid, the effectiveness of the DL segmentation method needed to be verified compared to the conventional threshold value method, including naïve thresholding or Otsu’s algorithm. In this manner, the MI models were analyzed using the histogram-based thresholding methods and the DL-based unsupervised segmentation method to compare the accuracy of assessment in MI size. The results of this analysis showed that the DL-based segmentation method was the most appropriate for evaluating myocardial viability and perfusion.

The aim of this study was to conduct a quantitative assessment of myocardial perfusion using the segmentation method. The DL-based unsupervised segmentation method for the assessment of MI size improved the accuracy of evaluation results compared to the histogram-based thresholding methods. The automatic quantitative assessment of the PET/SPECT polar map could determine myocardial viability and perfusion in accordance with the characteristics of each radiopharmaceutical. The DL segmentation method is expected to contribute to the development of cardiac research.

## Data Availability

The data that support the findings of this study are available from the corresponding author upon reasonable request.
